# Emergence of precursor instabilities in geo-processes: Insights from dense active matter

**DOI:** 10.1016/j.heliyon.2023.e22701

**Published:** 2023-11-23

**Authors:** Klaus Regenauer-Lieb, Manman Hu

**Affiliations:** a WA School of Mines: Minerals, Energy and Chemical Engineering, Energy Engineering Discipline, Curtin University, Perth, 6151, WA, Australia; b Department of Civil Engineering, The University of Hong Kong, HK, 999077, Hong Kong Special Administrative Region

## Abstract

We present the hypothesis that investigation of precursor mechanisms to large scale instabilities, that have so far been overlooked in geo-processes, is possible. These precursor processes are evident in multicomponent materials, such as granular matter, when driven far from equilibrium on its microscale. The material is then classified as “dense active matter” with unexpected behaviour by non-local dissipation of internal energy releasing its dynamic incompatibility with the macroscopic gradients as self-excitation waves under external forcing. These instabilities are known in solid mechanics as flutter instabilities, nucleating at what is more widely known as an “exceptional point” in a variety of systems when two or more eigenvalues of the system coalesce. The common principle to connect processes at and across their characteristic scales is investigated using a minimalist formulation by coupling the scalar field variables of solid and fluid pressures in a compacting porous medium. We present a multiphysics generalisation of the phenomenon to the exciting findings of fluctuations with oscillatory exponential growth which nucleate at the exceptional point for inception of complex conjugate eigenmodes and propose a rigorous theory based on the extension of Onsager's theorem to non-local processes. Future work will need to compare model predictions to carefully designed laboratory experiments and expand the work to bridge the scale of the laboratory to the scale of field applications including design of new sensors tuned for detecting exceptional points preceding collapse of materials.

## 1 Introduction

The use of thermodynamic potential functions has allowed the development of rigorous methods for constructing constitutive equations that can accurately describe the interplay between multiple scales and types of physics. Onsager's reciprocal relationship [1] is a powerful tool forming the basis for many linear relationships between thermodynamic forces and fluxes. Onsager's theorem implies that a local equilibrium exists, where linear functions of thermodynamic forces are sufficient to restore equilibrium and explain the linear diffusion laws observed in countless experiments [2]. Application of Onsager's relationship to nonlinear processes encountered in continuum mechanics led Ziegler [3] to propose the orthogonality principle of the thermodynamic flux (i.e., the dissipative strain rate) to the thermodynamic force potential (i.e., the yield potential). The orthogonality principle ensures maximum entropy production of the dissipative process. The assertion of maximum entropy production for nonlinear processes is needed to enrich Rayleigh's “principle of the least dissipation of energy”, as used by Onsager, for nonlinear constitutive relations. This is included in the orthogonality principle which for linear relationships coincides with the minimum entropy production through the requirement of the minimum of the Lagrange Multiplier around the linear force-flux steady state [4]. The principle of minimum entropy production can therefore be understood as a special class of the more general principle of maximum entropy production which also covers nonlinear constitutive behaviour of stressed solids. Another consequence of linear thermodynamic force-flux relationship is that there is no uncertainty about the magnitude of the diffusive operator and the results are unique because the variational maximum and minimum bounds of entropy production coincide [5].

However, real materials have microstructural randomness implying uncertainty in material parameters. In solid mechanics, the Voigt and Reuss bounds are well-established methods for estimating the effective elastic moduli of composite materials [6]. These bounds are based on the assumption of perfect alignment of the constituents in series or parallel, respectively. However, real materials exhibit microstructural randomness, which leads to uncertainty in their effective properties.

Entropic uncertainty principles offer a more general approach for bounding the effective elastic and dissipative properties of materials. These principles are derived from the variational bounds of entropy production extending the thermomechanics theory [3] by the bounds of entropy production (a rate quantity) in terms of the square of any irreversible current in the system [7]. This formulation also takes into account the current state of the configurational entropy of the system. The entropic bounds are applicable to both linear and nonlinear materials, and they can be used to assess the impact of microstructural randomness on the material properties.

The entropic bounds provide a number of advantages over the Voigt and Reuss bounds:

1. They are more general and can be applied to a wider range of materials.
2. They take into account the configurational entropy of the system, which provides a measure of microstructural randomness.
3. They are applicable to both linear and nonlinear materials.
4. They can be used to bound dissipative properties as well as elastic properties.

The extension of entropic bounds to dissipative processes is, however, a challenging task requiring accurate assessment of the internal dissipation processes and their irreversible currents. However, it has the potential to provide a powerful framework for understanding the behaviour of dissipative materials. The entropic bounds can be used to study multiphysics reaction-diffusion processes under non-isothermal conditions. They can also be used to investigate the behaviour of materials under extreme conditions, such as high temperatures and pressures. The use of entropic bounds is a promising new direction in the field of solid mechanics. These bounds have the potential to improve our understanding of the behaviour of materials and to lead to the development of new materials with improved properties.

We focus in this work on building the foundations for robust entropic uncertainty principles by calculating the irreversible currents in dense active matter for non-linear cases. In this case the assumption of linear force-flux relationships is no longer justified and there remains an uncertainty on the magnitude of the diffusion operator whether one considers an isentropic or isothermal microreversible cycle to evaluate the diffusion property [8], [9]. Ziegler's Thermomechanics approach assumes strictly isothermal condition making it impossible to investigate the entropy production of Thermo-HydroMechanical-Chemical (THMC) reaction-diffusion processes.

We have proposed an extension to relax isothermal conditions [10] for deformable porous materials. Without restricting its applications, the extension has been illustrated using the critical state line constitutive model with a modified Cam-Clay plasticity model [11]. This yield envelope is often used for modelling the behaviour of materials that can internally compact or dilate leading to macroscopic non-associated behaviour. For non-associated cases the strain rate is not orthogonal to the proposed yield surface in the true effective stress space. This apparent contradiction to Ziegler's orthogonality principle can be alleviated when recasting the yield envelope in the dissipative stress space where orthogonality holds, [10] under the restrictive assumption of local equilibrium.

In the field of continuum mechanics, the concept of dissipative stress was originally introduced as a systematic approach to constructing critical state line models for non-associative materials in three dimensions [12], [13]. These comprise granular materials that show the remarkable property of changing the volume when sheared. This dilatancy property will later be related to a dense active matter phenomenon which enables a coordinated pumping of fluids out of the sheared assembly of particles [14]. The physics causing this unusual material behaviour will be the main topic discussed in this article for which we use a thermodynamic approach to simplify the equations. It is therefore useful to compare the thermodynamic approach to the classical formulations in plasticity theory.

The thermodynamic approach eliminates the need for plastic potentials and replaces them with a dissipation potential. We propose that the dissipative stress approach also provides a promising solution to the long-standing problem of objectivity in materials that undergo rigid-body rotations during infinitesimal time increments. Objectivity requires that the state of stress and strain remains invariant under rotations of the coordinate system. This is particularly important for systems experiencing large deformations, as demonstrated by experimental studies [15].

Several objective co-rotational frameworks have been proposed as material reference frames that rotate with the material. These frameworks are well-suited for small deformations, but they break down under finite strain, leading to spurious oscillations or a hyperbolic response. This issue can be addressed by employing logarithmic strain measures based on the Hencky tensor, which have been shown to be accurate for non-associative materials [16]. The approach has also been demonstrated to provide an energetically consistent framework for simulating non-coaxial elasto-visco-plastic deformation at large strains and rotations [17].

The dissipative stress approach offers an alternative perspective on objectivity. Instead of explicitly seeking consistency with thermodynamic principles, it is directly based on the thermodynamic potential functions. Specifically, the dissipative stress is defined as the stress responsible for energy dissipation in the material. It is obtained by taking the partial derivative of the dissipation potential with respect to the plastic strain rate. The dissipative stress complements the generalized stress, which is calculated by taking the partial derivative of the Helmholtz free energy with respect to the plastic strain. The rate processes based on the current state of the material are hence characterised by the dissipative stress and the microstructural state at a given plastic strain is characterised by the generalised stress. In this formulation, the thermodynamic force used in Onsager's theorem is defined by the sum of the generalized stress and the dissipative stress.

The generalised stress includes irrecoverable internal elastic stresses - also introduced as stored plastic work or frozen elastic energy [18] - as these processes upgrade the Helmholtz free energy of the material. The dissipative stress is the stress that is lost as heat or the flow of matter when the material is deformed. Note, that this thermodynamic concept complements the geometrical interpretation of entropy as disorder. The formulation based on the entropy production allows separation of the dissipation potential function from the free energy function. The configurational entropy must appear in both the Helmholtz free energy and the dissipation potential function.

We believe that the dissipative stress approach has the potential to make significant contributions to the field of continuum mechanics. It provides a rigorous and consistent framework for addressing the challenges of objectivity and energy dissipation in materials undergoing large deformations. We emphasise that the approach requires, however, first accurate description of possible bifurcations from dissipative processes at microstructural level (the subject of the present work) and encourage further research in this area to explore the full potential of the dissipative stress approach.

In this contribution we consider an approach that is applicable to cases where Onsager's local equilibrium assumption potentially breaks down and the non-locality resulting from dynamic microphysics interactions needs to be considered explicitly. This is the case when dynamic microstructural processes are involved at a different pace than the bulk deforming ones and inclusion of, for instance, angular momentum from internal rotations needs to be considered. When including such small scale processes it is often found necessary to consider processes at grain scale to explain shear band thickness [19]. Modern computational models of localisation phenomena therefore include information from the micro-structure to enrich constitutive models for modelling the width and evolution of localisation phenomena in multiphysics-coupled applications [20]. When including such microphysics processes it does not necessarily imply that Ziegler's and Onsager's local equilibrium assumption is fulfilled.

At present we lack, however, an extension of Onsager's work to incorporate the important role that micro-structural processes play in the development of new constitutive relationships for multiphysics and multiscale coupling. For robustness such an approach ideally steps out of Onsager's local equilibrium Ansatz and establishes a macroscopic prediction of the curvature of the entropy function (zero for linear force-flux relations) from a perspective of the microscopic interaction as proposed by Jaynes [21]. This contribution follows Jaynes's line of thought enriching it by recent insights from the statistical behaviour of dynamic fluctuations in growing interfaces [22]. We conclude by proposing a new theorem based on Onsager's assumption of micro-reversibility applied to Onsager's off-diagonal cross-diffusion coefficients that are identified to characterise the non-locality of microstructural work neglected in the original formulation.

## 2 The need to extend Onsager theorem for excitable media

Onsager explored how the gradient of a generalized thermodynamic force $J_{i}$, which is created by a difference in thermodynamic potential $X_{j}$ (such as thermal, hydro, mechanical, or chemical), is related to the generalized flux, represented by laws such as Fourier (T), Darcy (H), Fick (C), and solid matrix pressure (M) diffusion. These laws are defined by diffusion coefficients that are associated with their respective processes. Onsager's theorem has stood the test of nearly hundred years of experiments for cases where local equilibrium applies also allowing for cross-effects between the different THMC processes.

(1) $$J_{i}=-\sum_{j=1}^{N}L_{ij}X_{j}$$

The Onsager diffusion operator $L_{ij}$ for linear THMC processes occurring in a porous geomaterial is:

(2) $$J_{i}=-\left[\begin{array}{cccc} L_{TT} & L_{TH} & L_{TM} & L_{TC}\\ L_{HT} & L_{HH} & L_{HM} & L_{HC}\\ L_{MT} & L_{MH} & L_{MM} & L_{MC}\\ L_{CT} & L_{CH} & L_{CM} & L_{CC} \end{array}\right]\;X_{j},$$

where the Onsager thermodynamic probability statement [1] of the relaxed system (non-equilibrium steady state) is defined by:

(3) $$L_{ij}=L_{ji},(\mathrm{for}\;i\neq j)$$

It is well-known that the original Onsager reciprocal relation does not consider the fast dynamic evolution stemming from microscopic interactions of cross-coupled processes and the coefficients $L_{ij}$ are under the assumption that the system evolves near local equilibrium.

**Figure 1:**
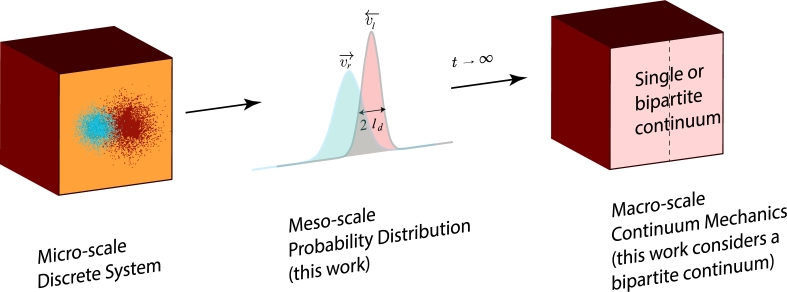
We use Girsanov's Theorem to obtain a meso-scale probability distribution of the stochastic processes of micro-scale discrete mass exchange processes cast into generalised thermodynamic diffusion processes at meso-scale. Inside a nucleating compaction band, for instance, these microprocesses are pore fluid flow and solid matrix compaction. Small fluctuations of Brownian-style motions of the micro-scale solid and fluid mass elements can lead to localised departure of the background porosity. For this to occur mass conservation implies that drift velocities *v*_*l*_ and *v*_*r*_ of the pore fluid and the solid skeleton must attract each other and be of opposite sign. This process results in a localised compaction of the matrix (see text). Such a situation translates at the macro-scale continuum mechanical level into a situation where the solid and fluid cross-diffusion processes cannot be eliminated [25]. We develop in this work an extension where we propose for such cases an alternative reference state defined by an additional time-scale of dynamic equilibration to two skew symmetric cross-diffusion coefficients. These coefficients are obtained from an integration of the meso-scale processes explicitly honouring the non-local gradient terms that carry the bipartite information from the microscale, averaged over the mesoscale, into the large scale continuum formulation.

We consider here interactions that may lead to large deviations from the local equilibrium state stemming from processes tied to the dynamics of the non-local, nonlinear cross-couplings at a meso-scale (see Fig. 1). As a particular example we discuss meso-scale processes causing internal mass exchange between solid and fluid components of a macro-scale porous system. The classical assumption is that there is no time-dependent mass-exchange and thus the two phases can be treated independently and be lumped together in a single macro-scale continuum. This convolution (of non-local processes) uses the simplified assumption of local equilibrium which in the absence of thermal forces implies that mass conservation is adhered to, i.e. a volume flux of fluid components is instantaneously replaced by an opposite flow of solid components. This assumption leads to the definition of Terzaghi's ‘effective stress’ (e.g., in 1-D an effective pressure $p^{\prime }=p_{s}-p_{f}$, where $p_{s}$ is the bulk solid pressure at equilibrium - and $p_{f}$ the equilibrium fluid pressure) used for definition of a single macro-scale continuum. Such processes therefore imply local equilibrium where Onsager's theorem applies and non-local fluid and mass exchange processes in the porous medium can be reduced to a local formulation.

The situation changes, however, drastically if we allow, in addition to the above described processes, internal mass exchange processes where a fluid component can, through a reaction, precipitate into the solid matrix or a solid can dissolve into the fluid phase. Another prime example is a granular system where the interaction of particles leads to a dilatancy phenomenon in a shear band. A similar case may be considered, where internal micro-cracking of the solid matrix allows for additional porosity, and fluids are attracted to these microchannels and have additional capacity to flow. Again these mass exchange processes are necessarily non-local and driven by the gradients of the thermodynamic forces, i.e., staying in 1-D, by the gradients in the solid pressure and the fluid pressure. We argue that these processes can be described by a non-local macro-scale continuum presentation, where in addition to the standard diffusion processes, described by the diagonal of the Onsager matrix, the non-local cross diffusion processes can play a significant role. This is the case when the solid pressure term triggers a non-local fluid flux and the fluid pressure term a non-local solid flux of opposite direction. Non-locality of the cross-coupling is introduced by the gradient terms which imply regularisation of the source terms over a diffusion length scale defined by the convolution of the two diffusion profiles in Fig. 1 over a common length scale $2l_{d}$. This convolution can incorporate positive feedback between the two processes leading to excitation between the pressure terms of two phases in the form of a non-local oscillation of an excitable medium. The objective of this contribution is to investigate under which conditions we can extend Onsager's approach to such excitable media.

In our previous work, we interpreted Onsager's approach as an entropic reference frame for the definition of dissipative properties under local equilibrium conditions [10]. The notion of an entropic reference frame is new and there is still some debate about its objectivity. One may argue that it is objective because it is based on the entropy of the system, which is a fundamental quantity in physics. Entropic uncertainty principles can be proven for linear force-flux relationships [23]. A thermodynamic objective reference frame can therefore be reliably defined by the thermodynamic potential functions of Ziegler's thermomechanics approach. This can be cast into Onsager's constitutive operator by properly identifying the dissipative stress as the appropriate thermodynamic force. The local equilibrium assumption may, however, not be well-suited for highly nonlinear interactions and non-local elastic rotations in excitable media such as granular matter. A rigorous derivation for nonlinear cases (as in our case) remains a serious, challenge although some progress has been reported recently [7].

In this paper we propose an additional dynamic thermodynamic reference frame for excitable media by making use of an entropic dynamic regularisation technique. This new reference frame applies to cases where the positive feedback between the microprocesses can nucleate excitation waves. The reference frame is defined by dynamic renormalisation of dissipative processes in the excited domain. We will conclude this paper by showing an example of dispersive, dissipative (flutter-type) excitation waves that propagate at constant wave-group velocity through the material. The waves nucleate for critical conditions of cross-diffusional multiphysics coupling across scales, upon non-local micro-reversibility of the microstructural processes, if such a non-local equilibrium exists. The new objective reference frame defines a possible deviation to Onsager's stable non-equilibrium macro-state and considers the growth of microstructural networks that can lead to a different outcome of the macrostate based on the extension of Onsager's theorem proposed by Casimir [24].

As a level of abstraction of Onsager's micro-reversibility - in the context of non-local continua describing microstructural work - we define non-local micro-reversibility as a perfectly coupled (diffusion controlled) linear combination of isothermal and isentropic thermodynamic loops in the temperature-entropy (T-S) space that either produce mechanical work (clockwise T-S loop, e.g., Carnot engine) and increase the entropy of the non-local continuum or require work to upgrade the entropy (negative entropy production in a counterclockwise loop; e.g., Carnot refrigerator). We refer in the following to these non-local processes as positive or negative entropy producing micro-engines. We propose that tight coupling (one micro-engine cannot run without the other) between the two micro-engines defines the new non-local equilibrium condition leading to the emergence of excitation waves.

**Figure 2:**
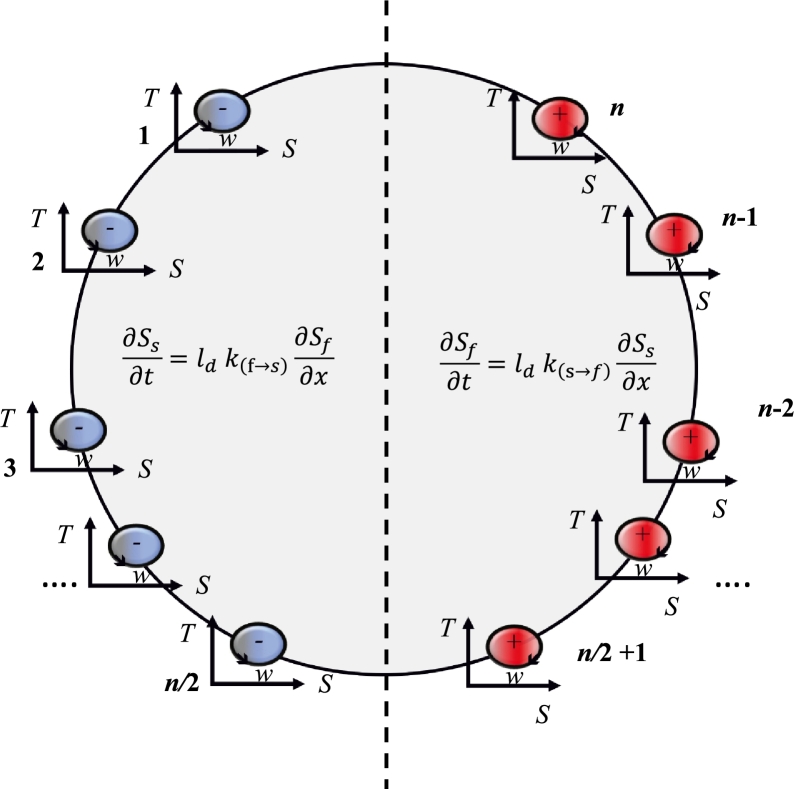
The concept of the minimal free energy landscape of tightly coupled dissipative processes. For the example shown in Fig. 3 the dissipative process is a non-porous dense solid that undergoes exothermic internal dissolution reactions under an applied thermodynamic force. The solid-fluid dissolution reaction is assumed to be maximally coupled as the dissolution reaction requires the generation of space through a dilatancy mechanism to allow for dissolution. Without the creation of space the dissolution reaction would be metastable. The relationship between mechanical deformation and chemical reaction is therefore cross-coupled. Mechanically induced dilatancy, within a gradient of solid entropy production $\frac{\partial S_{s}}{\partial x}$, drives the fluid flow and the fluid pressure generation, within a gradient of fluid entropy production $\frac{\partial S_{s}}{\partial x}$, drives the dilatancy mechanism. The matrix connecting the two cyclical micro-engines, i.e. the endothermic precipitation reaction and the exothermic dissolution reaction, is connected by elastic springs. The oscillators 1 to n/2 form the individual fluid parcels inside opening and closing pore spaces/cracks, the oscillators n/2 to n provide the work for the fluid pumping action. This example is known in the literature to result in a directional fluid transfer mechanism identified as a possible precursor phenomenon to macroscopic failure. In the earthquake community this excitation phenomenon is known as “dilatancy pumping” [51], [50].

**Figure 3:**
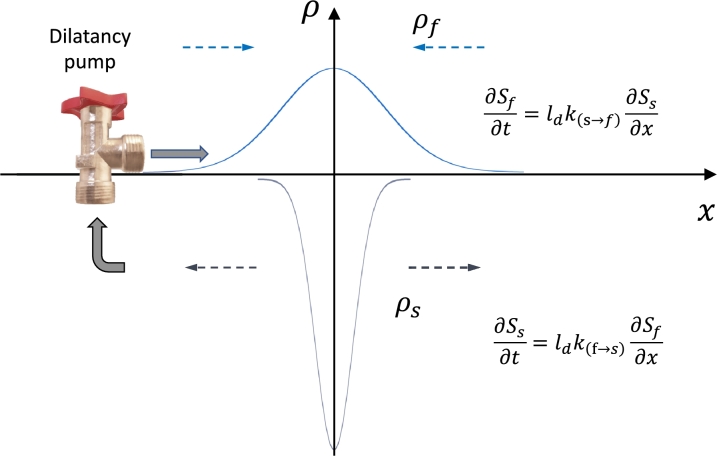
Solid-fluid coupling in a tight formation where fluids fill the space of the dilatant processes in the fault core. The dilatancy rate controls the pumping action of fluids into the region of highest dilatancy. The gradient of fluid pressure generation drives the mineral breakdown reaction, while at the same time the solid pressure gradient creates conditions for the dewatering reaction by dilatancy of the solid phase. The dilatant zone is described by a Gaussian probability function and generates space for the fluid, liberated by dehydration, and reduces the rock density *ρ*_*s*_. We identify the partial entropy production rates as *S*_*f*_, *S*_*s*_ for the fluid (chemical driven) and solid (mechanically driven) system. Both rate processes operate in balance within a common diffusion length scale *l*_*d*_ which multiplied by the forward and backward reaction rates $k_{\left(\text{s}⇌\text{f}\right)}$ yield the dynamic equilibrium of coupled positive-negative partial entropy production of solid and fluid phases. The nonlocal nature of the positive-negative coupling between the solid and fluid partial entropy productions over the length scale *l*_*d*_ stems from opposite mass fluxes of the two phases.

For illustration purposes, without limiting the generic abstraction, we consider the dissolution-precipitation reaction during compaction of a perfectly tight (no initial porosity) solid, such as in metamorphic reactions [26]. The approach also describes less extreme situations of mechanically enhanced fluid flow in the compaction of naturally permeable sedimentary rocks but the extreme case of a tight formation is useful, as it highlights the point that breakdown of the solid phase and the fluid assisted deformation process is only possible with a minimum of two tightly coupled microphysical processes. The proposition in this paper is that there exists a common active dissolution-precipitation reaction front resulting from a convolution of coupled solid-fluid reaction shown in Fig. 1. We will show that this maximally excited zone propagates with a constant group velocity containing the velocities $v_{r}$ and $v_{l}$ around a dynamic non-local equilibrium diffusional length scale $l_{d}$ which we attribute to a cross-diffusion coefficient. The velocities $v_{r}$ and $v_{l}$ result from multiplication of the dynamic equilibrium diffusion length $l_{d}$ with the different dissolution-precipitation reaction rates $k_{\text{s}\to \text{f}}$ and $k_{\text{f}\to \text{s}}$ as shown in Fig. 3. The endothermic precipitation reaction consumes mechanical work *w* and has a negative entropy production while the exothermic fluid release reaction generates mechanical work and has a positive entropy production. The tight symmetric coupling between the two processes can be understood as a maximally excited dynamic state dissipating all available free energy (here simplified as serially connected elastic springs, i.e. Voigt upper bound, linking the symmetric oscillators in Fig. 2) resulting in a minimum of the free energy landscape driving maximum dissipation of the maximally excited tightly coupled zone. This maximum requires an even number of oscillators to allow a symmetric split between fluid and solid components creating the maximally cross-coupled gradients between fluid and solid components. We postulate that a non-local equilibrium diffusional length scale $l_{d}$ exists that, multiplied by the reaction rate, defines the non-local equilibrium exchange velocity $v_{l}$ and $v_{r}$ which govern the fluid release or precipitation under thermodynamic gradients $\frac{\partial S_{s}}{\partial x}$ and $\frac{\partial S_{f}}{\partial x}$ along the spatial coordinates *x*.

The implied symmetry condition is exactly what is expressed by the proposed extension of the Onsager reciprocal condition to non-local conditions. The fundamental difference of the proposed non-local equilibrium condition to Onsager's local equilibrium condition is that we postulate the existence of a maximally excited state which is not only defined by the spatial coordinate *x*, as in Onsager's local equilibrium, but considers the concept of space *x* in time *t*. Away from this location of maximum dissipation the entropy production reduces over a distance defined by the non-local (solid-fluid) cross-diffusion coefficients. Outside of the excited zone the system reaches the uncoupled states of fluid movement and solid deformation defined by the background process of solid (Stokes) and fluid (Darcy) self-diffusion of the background permeable formation. In the extreme case of compaction of perfectly tight formation (such as in metamorphic rocks) this background is close to zero and Onsager's approach offers no solution for enabling fluid flow.

Before coming back to the extension of Onsager's approach we summarise the current understanding of entropic processes in continuum mechanics that lead to oscillatory material instabilities called “flutter instabilities” [27].

## 3 The current understanding of entropic considerations in disspative porous media

The idea of regarding the dissipation as a potential function is due to Ziegler [28]. The methods have been made more rigorous by various researchers in Europe [29], [30], [31], allowing simple and efficient formulations for derivation of constitutive behaviour of nonlinear materials on the basis of just two potential functions, the Helmholtz-, or alternatively the Gibbs- [32] free energy and the dissipation potential. The necessity to extend Onsager's formulation for non-linear media such as mechanical continua has led to a heated debate with Truesdell [33] leading the criticism. Truesdell suggests that Onsager's approach is not based on a robust upscaling of statistical mechanics. This criticism has been fully refuted [34]. The main point for the harsh criticism presumably is that the approach is based on the local equilibrium assumption that does not take into account the explicit non-equilibrium and non-local interactions between the particles in the system. These interactions can evidently violate local reciprocacy requesting further development of the theory which is the subject of the present work. Although the approach proposed by Jaynes [21] was recognised as a path forward the necessary insights from statistical mechanics have only been developed recently and are still part of ongoing research. A key problem which prompted criticism of the proliferation of Onsager's proposition as “Onsagerism” [33] lied in the lack of a consistent framework that rigorously identifies the appropriate thermodynamic forces and fluxes which is a non-trivial task and led to the proposition of a “rationale thermodynamic” approach. In an earlier communication [10] we have discussed the problem of properly acknowledging the role of internal elastic stresses in the microstructure inside a deformable medium such as in a non-associated modified Cam-Clay material. It has been clearly demonstrated that the use of the Cauchy stress as a thermodynamic force is inappropriate and a dissipative stress has to be used to define a dissipation potential function on which the thermodynamic flux (strain-rate) is co-axial [12], thus satisfying Ziegler's orthogonality principle and still obeying local equilibrium condition.

In this contribution we step further away from the local equilibrium assumption and explore the proposal of Truesdell [33] and Jaynes [21] to investigate the role of internal microstructurally stored energy, which is now well recognised in the theory of continuum thermomechanics [30]. Onsager's proposition is linked to the recent insights developed through the fluctuation theorem [35] by considering the entropy production of meso-scale processes underpinning the role of stored plastic work. Before upscaling the microstate processes into a meso-scale probability distribution (see Fig. 1) we first review the entropic considerations from the macro-scale perspective.

## 4 Acceleration waves and flutter instabilities in deformable solids

In this macro-scale view the release of stored energy from tightly coupled particles can cause dynamic incompatibilities to the existing internal stress gradients and lead to acccelerations that subsequently are imposed to the interface of the affected particle assemblies. Hill [36] conceives “acceleration waves” as particle acceleration and jumps in the spatial gradient of velocity, the velocity itself being continuous. The approach assumes single continuum constitutive laws and as the resulting jump in the gradient of velocity entails a jump in strain rate around these particle assemblies it also leads, via the material law, to a jump in stress rate. An acceleration wave can be interpreted in the sense of Hadamard [37] as an isolated geometric surface that moves relatively to the material and across which instantaneous material variables are momentarily discontinuous. This work has set the basis for the development of localisation instabilities as a standing acceleration wave of zero wave speed [38]. We recap briefly the key development:

Acceleration waves are generated by advecting the jump condition at a constant relative velocity of magnitude *c* along a wavefront moving through the material in direction *s*. Following Hill [36] we define the true traction (load vector per unit current area) exerted across the wavefront by the material on the side into which the normal points as **F**:

(4) $$\left[\overset{˙}{F}\right]=-c\left[\frac{\partial F}{\partial s}\right]$$

where following Hadamard [37] the square brackets indicate the jump condition $\left[\Omega \right]=\Omega -\Omega_{0}$ for any variable **Ω** where $\Omega_{0}$ is the background value. If [Ω] is nonzero a singular surface (wavefront) exists with Ω as the singular variable. All internal variables and the acceleration across the wavefront are constrained by:

(5) $$\left[\rho \overset{˙}{v}\right]=\left[\frac{\partial F}{\partial s}\right]$$

Combining Eq. (4) and (5) we obtain

(6) $$\text{[}\overset{˙}{F}\text{]}+c\text{[}\rho \overset{˙}{v}\text{]}=0$$

Substituting $\overset{˙}{v}=-c\frac{\partial v}{\partial s}$ into Eq. (6) we obtain

(7) $$\text{[}\overset{˙}{F}\text{]}=c^{2}\left[\rho \frac{\partial v}{\partial s}\right]$$

The acoustic tensor **Γ** is defined by

(8) Γ=n⋅C⋅n

The eigenvalues of **Γ** divided by the mass density represent the squares of the acceleration wave propagation speed in the direction of the unit normal vector **n** which in terms of acoustic tensor implies that:

(9) $$\Gamma \frac{\partial v}{\partial s}=\rho c^{2}\frac{\partial v}{\partial s}$$

Dynamic system stability in the sense of Hill [39] is hence achieved by real valued eigenvalues of the acoustic tensor thus determining the squared speed of the acceleration waves. For the special case of where the eigenvalue is zero, a standing wave is predicted which is then interpreted as a criterion for localisation instabilities.

(10) det(Γ)=0

In non-associate materials the eigenvalue problem can, however, become complex conjugate (in the sense that the roots of $c^{2}$ become complex conjugate) leading to the phenomenon of “flutter instabilities” or ill-posed solutions for vanishing wave-length perturbations [40]. The term “flutter instabilities” was introduced by Rice [27]. For a symmetric acoustic tensor **Γ** all roots of $c^{2}$ are real, however, for cases where the acoustic tensor is asymmetric the possibility of flutter instabilities was predicted given critical constitutive parameters. The flutter instability is often ill-defined in the literature because there are different ways to define it either through the acoustic tensor or the constitutive operator. We prefer the acoustic tensor definition [27] for a properly defined objective co-rotational or entropic reference frame as discussed earlier. The reason for this choice is that the constitutive operator, defined as a tensor that relates stress and strain-rate in a material, is often derived from laboratory experiments. Consequently, the constitutive laws are not necessarily strictly derived from objective co-rotational thermodynamic consistency considerations and there can be inconsistencies in the definition. In the context of acceleration waves complex roots of $c^{2}$ imply coupled oscillations or growth/decay modes in the material system. Although the theoretical reasoning for flutter-type material instabilities is straightforward, the phenomenon is still not completely understood [41], partially because it is derived from an abstract macroscopic concept which precludes insights into the fine-grain processes driving the occurrence of flutter instabilities. The instability was interpreted as a precursor phenomenon for strain localisation at zero wave velocity [42]. The real mechanical meaning of flutter has been attributed to an oscillatory motion of material particles, however, it was conceded that the mechanical interpretations are still difficult to assess [41]. Aside from these theoretical advances in understanding the flutter phenomenon the original comment by Rice [27] that “no specific case exhibiting this kind of divergence has been discussed” held true for a long time concerning experiments with non-associated frictional materials where stored plastic work plays a significant role for history dependent material behaviour. However, this has changed recently.

Numerical experiments with granular media allow a more detailed evaluation (from the microscopic perspective) of the coupled oscillations [43]. These observations constitute the first experimental proof for the flutter type instability. Molecular dynamics simulations of frictional granular assemblies revealed coalescence of real valued eigenvalues into exponentially growing complex conjugate modes at a critical applied strain. The evidence for a new micro-mechanical mechanism was argued to be crucial for the understanding of plasticity and failure in frictional materials. The authors also argue that the observation potentially offers a new avenue for understanding the physics of earthquakes as the mechanism allows self-amplification of small, long-wavelength perturbations. It was, however, unclear how the mechanism can operate in the context of the rich multiphysics of the Earth and its faults. We will address this point in the current contribution.

From a mathematical point of view the phenomenon of coalescence of eigenvalues is understood as an “exceptional point” [44] as the system becomes degenerate. At this exceptional point, not only do the eigenvalues coalesce, but their corresponding eigenvectors also coalesce. This behaviour can lead to significant consequences in the behaviour of the system where small changes in the system's parameters can cause rapid and drastic changes in the system's response. The relationship of exceptional points and instabilities is now well established in many physical problems [45]. The effect of coalescence of complex eigenmodes at the exceptional point is best studied in optics [46] where the exceptional point is identified as a degenerate eigenstate where the two eigenvectors merge into collinear eigenvectors with a divergent maximum. In geophysical fluid dynamics the coalescence of complex eigenmodes has, for instance, been identified as a trigger for the nucleation of exponentially growing modes at the onset of Rayleigh-Bénard convection and thermal Rossby waves [47].

The exceptional point (EP) phenomenon has, to the best of our knowledge, not yet been described in solid mechanics, except for the notion of flutter instabilities, which have not been explicitly attributed to EP theory. The EP bifurcation is different from the classical approach to instability, in which a bifurcation occurs when the constitutive operator becomes singular and one of the eigenvalues becomes zero. These bifurcations can occur at the critical point in conservative systems where Onsager's local equilibrium assumption holds.

EPs are spectral singularities in the parameter space of a system in which at least two or more eigenvalues, and their corresponding eigenvectors, simultaneously coalesce. This spectral collapse is a peculiar feature of non-conservative systems that exchange energy with their surrounding environment. EPs have been a hot topic in optics and photonics, and have matured into a robust theory that has allowed the development of new exotic laser generations with ultra-powerful flashes of light [48]. EPs have also been discovered as a new type of instability with the potential to cause extreme events.

We have proposed a non-local Onsager formulation that describes a non-conservative system due to the possibility of strong non-local interactions with the environment (large cross-diffusion coefficients). We postulate that poro-mechanical or granular systems should also exhibit EP instabilities. The above discussed experimental proof of this new phenomenon in granular matter [43] was not referencing the EP phenomenon but the EP point was accurately described to be encountered at a critical strain. At the EP point a pair of complex conjugate eigenvalues was found to be born, with four solutions of the form exp⁡iωt to the linearised equation of motion. One pair induces an oscillatory (flutter) instability with exponential growth, and the other pair an exponentially decaying oscillatory solution. The authors argue that the spectral collapse of small long-wavelength perturbations into a short sharp pulse through the mechanism of self-amplification of the excited granular medium may be a precursor mechanism to large scale failure.

The novelty of the EP phenomenon in dense active matter and the potential application to an earthquake source mechanism is beyond the scope of this contribution. A discussion of the physics of the EP in the context of classical bifurcation theory will be the subject of a forthcoming contribution, and the expansion to the multi-scale phenomenon of an earthquake is available as a short note [49].

## 5 Exceptional points turn passive- into active matter

In the following we propose that the described mechanism involving self-amplification of small-wavelength perturbations at the exceptional point can be further investigated by the micromechanical interactions responsible for the nucleation of growing interfaces called “active zones” [22]. We have already discussed micromechanical experiments demonstrating the nucleation of complex eigenvalues at the exceptional point here interpreted as flutter instabilities of non-associated critical state line models. The self-excitation phenomenon relies on the capacity of flutter instabilities to harvest energy from small, long-wavelength perturbations [43] thus preparing the system for failure. In a mechanical system this low amplitude long wavelength growth mode could be, for instance, the lowest eigenmode for a buckling problem of the system. For the fluid dynamic problem we have discussed the onset of Rayleigh-Bénard convection where equivalent fluid dynamic “flutter instabilities” coalesce at the exceptional point and lead to exponential growth of the Rayleigh-Bénard wavelength [47].

In the above examples the exceptional point acts as a transition point where the microstructural interactions transition from a passive state into an active, excitable state by harvesting energy from small perturbations out of their environment. A much discussed problem for such micromechanical coupling is the role of fluid redistribution through solid-fluid coupling in the tight earthquake source region where solid deformation is required to allow microscopic pore space opening (dilatancy) for the passage of fluids [50]. The physics of dilatancy was first investigated in the context of dynamical properties of media composed of ideal smooth particles in a high state of agitation through the dynamical theory of fluids [14]. The dilatancy concept was interpreted to be a possible nucleation mechanism for seismic faulting through “dilatancy pumping” [51]. The term “dilatancy pumping” was originally coined to describe the unknown mechanism of an experiment by Reynolds [14] in which a 3.4 liter rubber bag of sand and water sucked in about half a liter of water during the deformation of the sand bag. Reynolds [14] argues that “We have thus an instance of a medium transmitting waves similar to heat-waves and causing force between bodies similar to the forces of gravitation and cohesion..... More than this, by the separation of the two sets of grains there would result phenomena similar to those resulting from the separation of the two electricities.” This remarkable intuitive insight into the micromechanics of the experiment has yet been unproven. The relevance of the dilatancy pumping mechanism for regional-scale fluid transfer through microcrack dilatancy operating at high (100s of MPa) stress levels adjacent to active fault zones is also still uncertain [52]. The above discussed physics of excited media, that can nucleate at the exceptional point, may help in understanding the role of fluid redistribution in a deep and tight crustal environment where there is no natural permeability. The crucial question is whether it is possible to turn a passive into an active medium capable of pumping fluids through the fault zone.

Fig. 3 casts the empirical concept of an excited process through coupling between fluid and solid processes into a theoretical model by proposing coupling between the partial entropies of the two processes. In a tight formation fluid and solid mass-movement must operate in a tightly coupled manner to allow fluid transfer through the solid rock. We describe the fluid phase by $\rho_{f}$ assumed to be described by a normal density function. The fluid phase of a hydrous mineral is assumed to be released by a mineral breakdown reaction when the source rocks move out of the stability regime of their minerals. The mineral breakdown reaction remains, however, metastable if there is no space for the fluid to flow into. Space can be created by the dilatancy process through the deformation of the source rocks, either by microcracking or through a ductile process called “creep cavitation”, both resulting in a narrow (several cm-wide) shear band with peak dilatancy at the fault core reducing to the porosity to the tight country rock away from the fault core [53].

The necessity for tight coupling of the partial entropies stemming from the solid-fluid mass exchange processes is illustrated in Fig. 3. The diagram illustrates the coupled process of uphill diffusing fluids (dilatancy pumping), released by exothermic dissolution reactions, and mechanical processes that drive an outwards diffusing corridor of dilatancy. A negative-positive coupled diffusion direction is mathematically equivalent to applying Casimir's extension to the local Onsager's relationship by imposing an odd symmetry of positive and negative cross-diffusion coefficients. In the following we will have a closer look at the non-local processes in excitable media (the closest classification of the phenomena in physics is “active matter”). For this we temporarily abandon the macro-scale continuum mechanics restrictions and upscale the microstructural interactions from a stochastic perspective to obtain the probability distribution of the microstructural interactions in a meso-scale diffusion formalism as shown in Fig. 1. By developing a non-local perspective of these microstructural processes that turn passive matter into active matter we will provide a bridge between the finding of complex eigenvalues at the macroscale through acceleration wave theory and the above-described micromechanical perspective of granular assemblies [43].

## 6 Dense active matter in bipartite systems

Topological interactions between multiple objects are associated with the growth of patterns in clusters such as in colloidal systems, artificial particles, polymer macromolecules, biopolymers, viruses, interacting proteins, groups of animals, and other living beings. Such systems are now classified as “active matter” which do not obey the classical equilibrium thermodynamic rules as they dissipate energy at the level of their microscopic constituents [54]. Active matter exhibits unexpected behaviour by local dissipation of internal energy under an external forcing. A prominent feature of active matter is the display of self-propelled motion and self-assembly which is so far mostly observed in soft matter and biological networks. However, recently it was shown that dense extreme active matter constitutes an important material class [55]. Dense active matter relies on the physics of internal mass-exchange processes which drive the evolution of the system. Dense extreme active matter is defined by two properties: Firstly, the magnitude of the thermodynamic force triggering mass flux is larger than thermal forces that tend to destroy the directional motility of mass movement; second the typical persistence time is larger than characteristic relaxation times of the system in the absence of activity [55].

In such systems the growth of microstructural interactions leads to the formation of interacting network structures that evolve over time, thereby significantly affecting the nature of their macroscopic behaviour. In polymers, for instance, the interpenetration of macromolecules creates physical networks to form entangled chains of macromolecules that significantly affect the final elastic property, rheology and its morphology [56]. The dynamic process of growth of networks of entangled chains may be considered by the growth statistics of an “active zone”. However, once the growth process is terminated the polymer chains can be classified as passive matter. In most cases we are only interested in the passive state and subsume the material modifications formed during the active phase into effective material constants.

However, in rare cases and for very short instances, flutter instabilities and their degenerate behaviour at the exceptional point can lead to large deviations that can take control of the macrosystem in the form of extreme events such as the phenomenon of rogue waves observed in oceans [57], [58], [59]. In most cases, however, flutter instabilities are precursors of stable modes, as in the classical localisation problem for zero acceleration wave velocity [38]. In this case cross-couplings relax so that their contribution can be adiabatically eliminated and a stationary pattern emerges, where Onsager's stable single continuum non-equilibrium macrostate wins (Fig. 1) and time evolution does not play a role. A particular motivation for this work is the interest in explicitly understanding the role of cross-couplings in materials as their role is to prepare the familiar stationary instabilities observed in porous materials, such as compaction bands or shear bands. In order to explore the role of these dynamic processes, that can develop before the conventional plastic limit in the form of flutter instabilities or divergence modes [60], we compare the classical thermomechanics approach to the thermodynamics approach for active matter.

We have already discussed the first approach relying on the physics of acceleration waves [36], [42], [61] which originate when the existing internal stress gradient is dynamically incompatible with the acceleration imposed by internal particle interactions [62]. The second approach is based on statistical mechanics and takes a closer look into the processes that lead to the violation of local equilibrium. The approach investigates the growth of clusters that form static and dynamic fluctuations in an “active” zone through a meso-scale analytical coarse-graining [22].

One of the most striking examples in this field is the direct transformation of an inert sand-pile into dense active matter through the application of shear strain [63]. This direct link between sheared granular media and active matter holds the promise of making significant progress in advancing our understanding of dense active matter theory. The fundamental driver of the active matter phenomenon lies in the particles within granular materials, which can engage in interactions through contact forces. These forces can be either repulsive or attractive, with their magnitude contingent on the inter-particle distances. The intricate interplay among these particles within granular materials can engender an array of phenomena, including fluctuations within particle groups, the formation of networks, and the emergence of long-wavelength instabilities such as shear band formation.

Granular matter research may therefore not only shed new light on precursor phenomena to large-scale instabilities but also presents unprecedented opportunities. The findings position granular media as an ideal platform for delving into the physics underlying the collapse of both natural and engineered structures. Research in this domain has already demonstrated that in the proximity of a potential collapse, the particle assembly is predominantly governed by the lowest eigenmode. This lowest eigenmode crucially determines the path along the energy landscape that particles follow [64], ultimately leading to the formation of shear bands. A promising future research direction therefore is to combine the theoretical prediction of this study with future experiments of sheared or compacting granular media. To this end we present an approach that allows upscaling of the directional motion of active particle assemblies along the path of the lowest resistant energy barrier which is well described by the growth dynamics of active zones.

### 6.1 Upscaling the growth dynamics of the active zone

In a pioneering paper an analysis has been proposed [22] to describe the dynamic development of such growing interfaces of the active zone network structures. The growth of these networks can be described by a nonlinear Langevin equation:

(11) $$\frac{\partial h}{\partial t}=\nu \frac{\partial^{2}h}{\partial x^{2}}-\frac{\lambda }{2}{\left(\frac{\partial h}{\partial x}\right)}^{2}+\eta (x,t)+k$$

where h(x,t) is the height of the stochastic growth of the active zone -in the above sheared sand pile example the directed motion of the particle assembly to achieve the minimum of the free energy landscape -, *ν* a diffusion coefficient for smoothing the interface and *λ* is the coefficient of the lowest order nonlinear growth term describing the dependence of growth on the slope of the interface. Kardar [22] emphasise that higher orders can also be considered but they do not contribute to the universal scaling relationship. η(x,t) is a noise term and *k* is a constant growth rate which is identified in later work to contribute to the positive average growth [65]. The equation captures an important universality class of stochastic growth of active zones which have been verified in many high-precision experiments.

We use a thermodynamic perspective for upscaling the stochastic interactions into an evolution of the probability distribution of the growth of the active zone. In this context the height of the stochastic growth, averaged over the noise for a two phase system, can be understood as being identical to the entropy production of the active zone [65]. The growth with time is found to obey a linear relationship:

(12) $$h(x,t)\equiv \overset{¯}{S}(x,t)=vt+\beta t^{\alpha }$$

By averaging over $\overset{¯}{S}(x,t)$, the entropy of the active zone can be evaluated as the mean-height of the stochastic growth of the active zone described by two divergent time scales of active zone growth. The first time scale is related to a characteristic velocity of internal mass exchange processes within the active zone *v*. A second time scale is connected to the growth of the width of the fluctuations controlled by a positive constant *β* and an exponent *α*. This term governs the size of fluctuations in the interface height, which can be derived from simulations of percolation networks [66]. This universal network growth relationship was confirmed by theoretical predictions [22]. The linear active zone growth is common to all active zones making the velocity *v* in Eq. (12) an important material parameter describing internal mass exchange processes in such systems. The approach is valid for a wide case of applications. In this paper we focus on understanding the dynamics of the active zone for the case of evolution of solid and fluid pressures to decipher the microphysics of Reynolds dilatancy pumping mechanism. We propose two coupled microstructural processes for which the probabilities distributions can be parameterised as Gaussian shaped fluid and solid pressure distributions. For this case the fluctuations defining the width of the active zone in Eq. (12) can be parameterised by a characteristic diffusion process through the length scale $l_{d}$ [67].

Before continuing with a discussion of the propagation velocity of the active zone we sketch very briefly the relationship of the following approach to the more recent developments in information theory leveraging on the well developed mathematical proofs for uncertainty quantification for the stochastic interaction of two distributions. The problem addressed in this paper is related to the well-known empirical Schrödinger bridge problem used in machine learning algorithms [68]. The bridge is defined as the most likely stochastic co-evolution between two probability distributions (see Fig. 1) given a prior stochastic evolution. The relationship to the work in this paper is that the bridge is defined as a stochastic optimal transport problem that deals with finding the most probable path for a diffusion process to evolve from an initial probability distribution to a final probability distribution in a given time interval. The approach is based on the gradient Ansatz of the Girsanov transformation which leads to a more general problem of the spread of information velocities given by a parabolic partial differential equation (*pde*) with a quadratic nonlinearity resulting from an upscaling of the Langevin equation (here Eq. (11)) into a paired Fokker-Planck equation (not discussed here, see Léger [69]) and a Hamilton–Jacobi–Bellman equation. The Hamilton-Jacobi-Bellman equation is nonlinear due to the presence of the squared norm of the gradient of some scalar field Φ(x,t).

(13) $$\frac{\partial }{\partial t}\Phi (x,t)+\gamma \nabla^{2}\Phi (x,t)+\frac{1}{2}|\nabla \Phi (x,t){|}^{2}=0$$

where *γ* is the diffusion parameter of the field variable. Using the Hopf-Cole transformation by exchange of variables (see Léger [69]) with exponential growth of decay characteristic, the co-evolution of the spread of information can be turned into two coupled linear ${\text{pde}}^{\prime }s$.

(14a) $$\frac{\partial }{\partial t}h(x,t)-\gamma \nabla^{2}h(x,t)=0$$

(14b) $$\frac{\partial }{\partial t}h^{⁎}(x,t)+\gamma \nabla^{2}h^{⁎}(x,t)=0$$

This bipartite coupled balance equation of the two reactive source terms and the diffusion mechanism forms the basis of the current non-local (cross-diffusion) approach. The problem in this work is, however, slightly more intricate than the above-mentioned optimal stochastic transport problem. We are primarily concerned with finding an evolution equation of $\frac{\partial }{\partial t}h(x,t)$ within the active zone that quantifies the shared information of the bi-partite coupled system across multiple scales [70]. The machine learning problem on the other hand is about finding a bridge distribution that satisfies the constraints and is closest to the initial distribution, by using a measure of the difference between the two probability distributions. Therein, the evolution equation is obtained by minimisation of the relative entropy which can be interpreted as the squared of the Euclidean distance [71] between the two distributions (Kullbeck-Leibler divergence).

The propagation of the “active zone” is, on the contrary, found to stabilise in a dynamically propagating network structure, based on the sharing of information across the tightly coupled bi-partite system interaction disregarding the initial constraints. Here we will first, only discuss coupling between two scales, using the dilatancy pumping mechanism as an illustration, but the problem will be later on generalised to an approach for multiple scales. The spread of information for each of these scales is characterised by their own reaction-diffusion equation (Eq. 14b) with their associated length scale $l_{d}$, independent of the initial conditions. In Fig. 2 we have proposed that a perfectly correlated nonlocal state can be used as an alternative to the Onsager reference frame for cases where the active zone plays a role. This correlated state can be understood as the optimum for a bipartite system with a maximum mutual information, i.e. the total amount of information that can be gained about the endothermic oscillators by measuring the exothermic ones across the dynamic equilibrium length scale $l_{d}$. We therefore suggest to use the extremum of the mutual information entropy rather than the relative entropy in the sections to come.

### 6.2 Propagation velocity of the active zone

In the classical single continuum approach the active zone does not exist and the reactions inside the active zone constitute, from the perspective of the macro-system, a violation of the local equilibrium assumption [62]. In the single continuum view the active zone radiates energy, through step functions, away from the sites of violation of local equilibrium in the form of acceleration waves. The question arises whether the velocity of these acceleration waves can be identified as the propagation velocity of the active zone which can be estimated by using considerations on entropy production as this quantifies the dissipation of the propagating wavefront.

In Fig. 2 we have defined an active zone in terms of mutual information meaning that both processes share the same information through the tight binary coupling. The free energy stored in the fluid saturated, non-porous solid rock mass can be released by the internal chemical reaction which needs to be activated by mechanical compression of the solid medium. In such a situation fluids present in the solid minerals are tightly coupled with the mechanical processes, i.e., an internal solid-to-fluid phase change reaction cannot occur without the accompanying crack opening. In this context the mutual information defines a limiting velocity of propagation of the active zone, and is understood as the velocity of the propagation of fluctuations triggering reactions through, e.g., release of stored energy reached under a critical load thus turning passive matter into active matter. Eq. (12) identified the velocity *v* as the characteristic velocity of the active zone within the diffusional length scale $2l_{d}$, regularised by minimising the free energy (Figure 3, Figure 2) through a constructive convolution of the partial entropic velocities of the two waves in Fig. 1. This velocity is related to the propagation velocity of the active zone characterised by the progress of the dissipative active zone.

We have pictured the dissipation in terms of the micro-engine analogy as a series of positive entropy producing micro-engines (Fig. 2) of e.g., the dilatancy cracking mechanism. The opposite directed flow of fluids is conversely resulting in an uphill diffusion thus upgrading the entropy and hence understood as a series of negative entropy producing micro-engines. Tight coupling implies that there is no master-slave relationship but both processes excite each other as in the Lorentz strange attractor. The strength and locality of the excitation controls the speed of the propagation of the active zone. The velocity is expected to be faster for stronger and closer interactions. The positive entropy producing micro-engines (dilatancy mechanism) propagate in the 1-D *vt* coordinate system ahead with a velocity $v_{r}$, as they consume some of the energy stored in the microstructure to contribute to the propagation, while the negative entropy producing micro-engines receive work from the microstructure and trail with a velocity $v_{l}$. This self-excitation is here interpreted to be the physical mechanism underpinning flutter instabilities $v_{\left(l,r\right)}$ of fluid and solid phases propagating to the left and right of the wavefront where $v_{\left(l,r\right)}<2v$. This implies a paternoster-type hydromechanical coupling between the propagation of void space and fluid migration of the dilatancy pumping mechanism. The wavefront velocity of fluid transport is thereby defined by the sum of the velocities in the active zone, here interpreted as being limited by the velocity of the acceleration wave. For expansion of this approach to a nested scale of THMC diffusion length scales, as discussed later, the propagation velocity is still a matter of ongoing research. It is thought to be related to self-organisation through a series of collective dynamical states at multiple hierarchical levels with an ultimate limiting velocity of the spread of information [72].

We have proposed a note on the possible extension for such multiscale hierarchical coupling of THMC processes [49]. Here we focus on the simpler hydromechanically coupled system which offers new perspectives through passive matter turning into active matter. In continuum mechanics, it is generally held that entropy increases in an isolated system. However, there are some cases where entropy can decrease, such as in the case of active matter, as discussed here for the case of the dilatancy pumping mechanism of the serpentinite dehydration problem in Fig. 3 interpreted as an active matter phenomenon.

The negative entropy production stems from turning the metastable serpentinite material (having reached the critical dehydration temperature) from passive matter into active matter by a mechanically imposed strain. Serpentinite will then compact up to 20% of its solid mass through dehydration into a normally distributed mechanical dilatancy cracking mechanism around the centre of the dilatant compaction band which has the highest porosity. This implies that the solid is flowing in the normal direction of decreasing pressure. The fluid is flowing, however, in the direction of increasing pressure. This is a non-equilibrium situation, and it leads to negative entropy production.

The entropy production of the active zone is, from a macroscopic point of view, negative because the work done by the solid is not being lost to the macroscopic environment. The reason for this is that the solid is doing work on the fluid, allowing it to flow against the pressure gradient. This upgrading of the fluid's entropy (negative entropy production) is compensated by an increase in the entropy of the solid. This type of negative entropy production is known to power microscopic machines.

In the light of the micro-engine analogy in Fig. 2 the engine that supplies the work for the uphill diffusion of fluids is the positive entropy production of the exothermic dissolution reaction. In the following we will derive explicitly the non-local entropy production, by first simplifying the problem into a hydromechanically coupled problem. This implies that we use Ziegler's extended thermomechanics assumption [10] for the thermal problem and ignore the thermal diffusion process. In other words we use an adiabatic thermal assumption and reference all cross-coupling material coefficients to the reference temperature. This allows us to upscale the non-local negative entropy production through consideration of a negative $L_{HM}$ cross-diffusion coefficient coupled to a positive entropy producing $L_{MH}$ coefficient. An alternative formulation to the non-local negative entropy cross-diffusion $L_{HM}$ is to introduce a “fictive” third entropy producing self-diffusing species in the macroscopic formulation [73].

By neglecting temperature feedback we have abstracted the multiphysics process to the classical dilatancy pumping mechanism discovered by Reynolds [14]. For this problem the configurational entropy allows a more straightforward evaluation of the entropy evolution. We note, however, that both approaches are complementary as the evolution of the configurational state does not give the process information of energy and mass exchange. Configurational entropy is a measure of the logarithm of the number of microstates that correspond to a given macrostate. It is thus directly related to the information entropy of the system. Following the thermodynamic configurational formulation for sand packs [74] we conclude that the source of negative entropy is associated with a coupled positive - negative evolution of configurational entropies during compaction of the solid matrix. The force driving the fluid into a negative entropy configuration actually originates from the positive entropy production in the solid matrix where an increase in the porosity is driven by the reduction of the Helmholtz free energy $S_{s}=-k_{B}(dF/dT)$ due to the exothermic dissolution reaction. $S_{s}$ is the total entropy of the solid matrix, $k_{B}$ the Boltzmann constant and *dF* the incremental change of the Helmholtz free energy.

For obtaining further information about the propagation dynamics of the positive/negative entropy producing excited zone we have to return to quantifying the entropy production of the dehydration process. The entropy considerations for the excitation waves describe the growth dynamics of active zones which we identified as the micromechanical analogues of the flutter instabilities derived from classical single continuum theories in terms of the complex conjugate roots of $c^{2}$ in Eq. (8) if non-associated constitutive behaviour is considered [42], [61], [60], [75]. We propose that a direct comparison between flutter instabilities and the $v_{\left(l,r\right)}$ velocities can be made as both are examples of how small fluctuations in one part of a system can propagate at a finite velocity through the system and have a large effect on the system as a whole. Although the macro-scale single continuum concept and the bipartite non-local mesoscale approach for the propagation of active zones are different theoretical concepts we can establish a closer link than the above illustrative example through the discussion of the role of entropy in both concepts.

The main difference is that in the macro-scale theory the violation of the local equilibrium is investigated by the assumption of a discrete Hadamard jump condition, whereas for the concept of the propagation of an active zone the jump condition is replaced by a diffusive length scale $2l_{d}$. We may therefore expect to get further insights into the physics of flutter instabilities from the latter theory. For this we investigate the entropy production of the active zone.

## 7 Derivation of the cross-diffusion equation for excitable bi-partite continua

**Figure 4:**
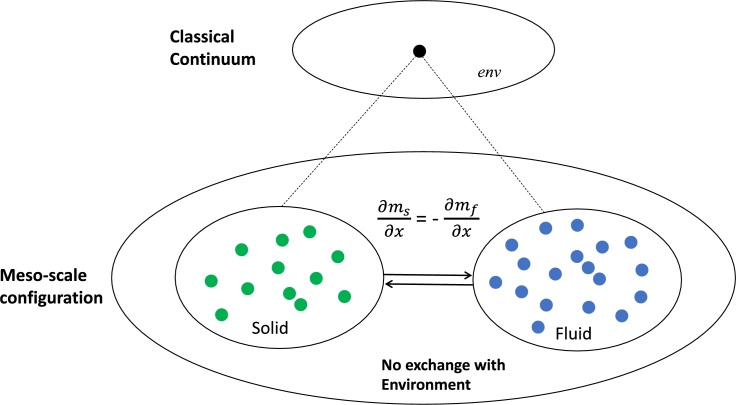
The *single-continuum* perspective and its connection to the local hydrodynamic *two-phase fluid/flow* perspective at steady state. The coarse-graining operation comprises a network of three continua. In the meso-scale configuration, the solid matrix (represented by green dots) is connected with the fluid phase (blue dots) through possible reactions (double arrow) where fine-grain mass/energy exchange processes are considered as a steady state system at local quasi-equilibrium scale with conservation of mass and energy. This allows the connection of the two phases to be considered inside the coarse-grained configuration as a single entity (conceptualized as a black dot). There is no additional dynamics considered by the interaction of the solid and fluid phases with their meso-scale environment.

We have introduced the rationale to use the mutual information entropy for deriving the evolution equation of an active zone comprising a finite subsystem of solid and fluid components that are pairwise connected and interacting over a diffusive length scale $2l_{d}$. The subsystems are embedded in a large scale macroscopic continuum, a fluid saturated porous medium in local equilibrium, capable of contributing or receiving entropy flow from the joint solid and fluid phases which constitute in Fig. 4 the environment $S_{env}$. This implies that in the zone that is not affected by the active zone the application of Onsager's theorem leads to Terzaghi's effective stress principle in the environment.

The entropy of mutual information between the solid phase and the fluid phase in the active zone now is defined as:

(15) $$S_{s,f}:=S_{s}+S_{f}-S_{env}$$

In the following we will test the proposition that the velocity of acceleration waves can be derived from the upper bound velocity of propagation of active zones 2*v*. This implies that the flutter velocities proposed by Rice [27] can also be derived from the meso-scale perspective as part of the physics of the propagation of active zones corresponding to the velocities $v_{l},v_{r}$. The proposition will be tested by a linear stability analysis to identify critical parameters followed by a numerical solution of the non-local reaction-cross diffusion equation derived from the above defined mutual entropy (Eq. (15)). The new reaction-cross diffusion equation is obtained by embedding the meso-scale active zone into a macro-scale system using a linear combination of entropies as discussed further below (Eq. (18)).

The key assumption is that the dynamic renormalization approach [22] describes a non-local equilibrium of the active zone. We interpret the gradient term (second term in Eq. (11)) as the key driver for the entropy production of the reactions. This term (and higher order terms) is used to calculate the additional bipartite mutual information entropy of the networks of tightly coupled micro-engines. The total entropy production can now be decomposed into the time-independent fluctuations characterised by a pre-factor $l_{d}$ (quantifying dynamic renormalisation of the diffusion controlled length scale of the entropy density towards which the system is equilibrating) and the two reaction rates $k_{\left(\text{s}⇌\text{f}\right)}$ describing the local production rate of mutual information between solid and fluid components of the subsystem triggering fluxes due to local gradients of the bipartite system in conjunction with the entropy exchange with their environments.

The foregoing discussion allows the introduction of higher precision of time resolution, required for describing the constitutive behaviour of an active (excitable) medium. We need a formulation that allows for a complete description of all fine-grained microstates and their evolution on at least two different time and spatial scales. In this contribution we only consider the pressure equation for two micro-states and two scales for illustration; an extension to multiphase materials and multiple THMC (and other) fluxes and forces is straightforward [62], [76].

### 7.1 Macroscopic single continuum view

We introduce the methodology by discussing the classical single continuum view of the macroscopic system (Fig. 1). This is defined by the long time-scale non-equilibrium steady state of the fluctuations of the time averaged irreversible entropy production which can be described by the local equilibrium assumption implying that the fluid and solid phases are in local equilibrium as shown in Fig. 4, i.e. no active matter phenomenon is considered.

The mutual entropy production of the single continuum macroscopic porous medium consisting of fluid and solid subsystems can now be evaluated. This is evaluated by applying the time derivative of Eq. (15) to the coarse grain continuum identified by a superscript *cg*.

(16) $${\overset{˙}{S}}_{s,f}^{cg}={\overset{˙}{S}}_{s}^{cg}+{\overset{˙}{S}}_{f}^{cg}-{\overset{˙}{S}}_{env}^{cg}$$

Since the single continuum macroscopic system assumes local equilibrium there is no entropy production and the mutual information ${\overset{˙}{S}}_{s,f}^{cg}$ is zero. If the macroscopic continuum is a closed system the marginal entropy production of the fluid and solid subsystems fully define the joint entropy $S_{env}$ of the porous solid-fluid medium. The local equilibrium assumption of the fine-grain mesoscale system implies that there is no entropy flux of the solid and fluid subsystems into or out of the mesoscale environment, i.e. the mesoscale is considered a closed system (Fig. 4). Coming back to the Terzaghi effective stress principle this also implies that the fluid and solid mass movements are fully uncorrelated states, i.e. there is no coupling between the deformation work of the solid matrix with the flow of the pore fluid [77].

This approach is used in Ziegler's classical thermomechanics theory. We have shown [10] that Ziegler's approach can be generalised through Onsager's microreversibility assumption into a generic reaction-diffusion formulation (relaxing the thermostatic assumption) if the far from equilibrium system is attracted to a steady state that is characterised by the adiabatic elimination of the cross-diffusion coefficients. Under this assumption the cross-coupling between different thermodynamic-forces and fluxes can be described simply by modifying the two self-diffusion coefficients of the solid and fluid phase which define the dissipation of the thermodynamic reference state through the multiplication of thermodynamic forces and fluxes.

### 7.2 Bipartite macro-scale continuum view

The above coarse-grained perspective neglects the dynamics of the fine-grained system as encountered in systems where reactions between fine-grain particles add additional local dynamics. With a coarse-grain assumption a complete analysis of all microstates and their dynamic configurations is therefore not possible and bias is introduced through limitation to local processes that have relaxed to their steady-state equilibrium in the coarse-graining analysis. In order to assess the bipartite-continuum system entropy production ${\overset{˙}{S}}_{\text{s,f}}^{bi}$ we follow the same approach as for the single continuum derivation but consider in addition the fine-grained processes and the interaction with their respective environments including the coarse grain environment.

From the bi-partite configuration perspective, we identify following multiparty mutual information entropies between coarse grain mappings (superscript *cg* and fine grain superscript *fg*)

(17a) $${\overset{˙}{S}}_{\text{s}}^{\text{bi}}=\overset{{\overset{˙}{S}}_{\text{tot }}^{\text{s}}}{\overset{︷}{{\overset{˙}{S}}_{\text{tot }}^{\text{s,fg}}+{\overset{˙}{S}}_{\text{tot }}^{\text{s,cg}}}}-\overset{{\overset{˙}{S}}_{\text{env}}^{\text{s}}}{\overset{︷}{\left({\overset{˙}{S}}_{\text{env }}^{\text{s,fg}}+{\overset{˙}{S}}_{\text{env }}^{\text{s,cg}}\right)}},$$

(17b) $${\overset{˙}{S}}_{\text{f}}^{\text{bi}}=\overset{{\overset{˙}{S}}_{\text{tot }}^{\text{f}}}{\overset{︷}{{\overset{˙}{S}}_{\text{tot }}^{\text{f,fg}}+{\overset{˙}{S}}_{\text{tot }}^{\text{f,cg}}}}-\overset{{\overset{˙}{S}}_{\text{env}}^{\text{f}}}{\overset{︷}{\left({\overset{˙}{S}}_{\text{env }}^{\text{f,fg}}+{\overset{˙}{S}}_{\text{env }}^{\text{f,cg}}\right)}},$$

A linear superposition of Eq. (17a), (17b)a and Eq. (17a), (17b)b leads to the multiparty mutual entropy of the entire system (Eq. (18)).

(18) $${\overset{˙}{S}}_{\text{s,f}}^{bi}=\underset{{\overset{˙}{S}}^{\text{tot }}}{\underset{︸}{{\overset{˙}{S}}_{\text{s}}^{\text{tot }}+{\overset{˙}{S}}_{\text{f}}^{\text{tot }}+{\overset{˙}{S}}_{\text{cg}}^{\text{tot }}}}-\underset{{\overset{˙}{S}}^{\text{env }}}{\underset{︸}{\left({\overset{˙}{S}}_{\text{s}}^{\text{env }}+{\overset{˙}{S}}_{\text{f}}^{\text{env }}+{\overset{˙}{S}}_{\text{cg}}^{\text{env }}\right)}}$$

**Figure 5:**
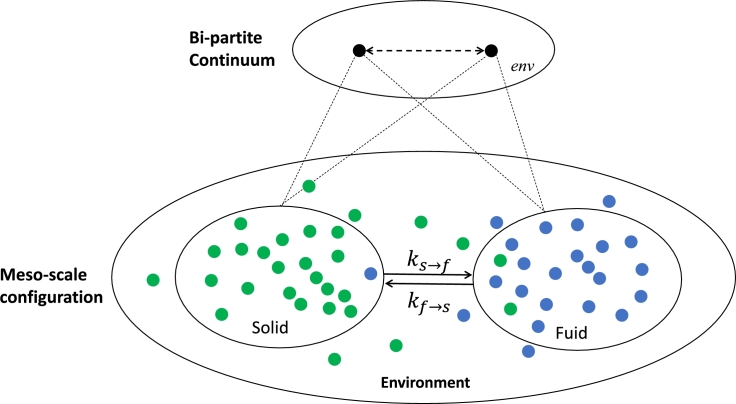
The *bipartite-continuum* perspective. The fine-grain bi-partite continuum perspective relaxes the assumption of steady state equilibrium of the local mass/energy exchange processes between the fluid and solid phase. Fine-grain processes are considered as an open system at local scale. Due to the open system assumption at fine-grain, two pairs of coupled diffusion processes need to be considered and the meso-scale is mapped into a bipartite continuum at coarse-grain scale.

This perspective allows embedding the active zone dynamics into the large scale continuum. The consideration implies that at the large-scale we cannot collapse the solid and fluid phases into a single representation but need to consider them as a bi-partite continuum shown in Fig. 5. The formulation applies to a generic system and is illustrated by the dissolution-precipitation case discussed in this paper. It encapsulates a wide variety of nonequilibrium reaction phenomena such as the propagation of flame fronts, aggregation of colloids and smoke particles, strain hardening due to dislocation entanglement, polymer chain entanglement, spreading of viruses, tumor nucleation and growth, etc. according to [22].

For evaluating the effect of the active zone on the bi-partite large scale system we need to specify the evolution equations for $\frac{\partial S_{s}}{\partial t}$ and $\frac{\partial S_{f}}{\partial t}$ describing the fine-grained dynamics of the dissipation potential between solid and fluid phases. For this we use the upscaling of the dynamics of the gradient term in the active zone of Eq. (11). This dynamics is characterised by the marginal entropies of the solid and fluid components depicted in Fig. 2.

(19a) $$\frac{\partial S_{s}}{\partial t}=l_{d}k_{\text{f}\to \text{s}}\frac{\partial S_{f}}{\partial x}$$

(19b) $$\frac{\partial S_{f}}{\partial t}=l_{d}k_{\text{s}\to \text{f}}\frac{\partial S_{s}}{\partial x}$$

We can now come back to the proposition in section 2 and interpret the diffusion length-scale $l_{d}$ introduced in Fig. 1 to define the entropy density towards which the active zone is equilibrating dynamically. This non-local equilibrium entropy density is the time independent state of the non-local, time-independent fluctuations that drive e.g. the fluid flow in the dilatancy pumping mechanism. The non-local, steady state of the width of the propagating active zone is found to be characterized by a diffusion-limited aggregation problem where all new particles are collected around the active region [78]. The reaction rate $k_{\text{s}\to \text{f}}$ is identified as the rate function for entropy production introduced from the fluid mass generation by dissolution and $k_{\text{f}\to \text{s}}$ the solid mass rate fucntion for the opposite direction. For thermodynamic consistency the two diffusion directions must have opposite sign implying that one of the phases features uphill diffusion. This leads to a skew symmetric Onsager matrix.

## 8 Extension of Onsager's approach for skew symmetry

Meixner [79] noted that the approach of Onsager needs additions when dealing with the thermal diffusion problem in the case of paramagnetic gases. If a magnetic field is applied the diffusion problem becomes anisotropic and Onsagers reciprocity approach needs to be extended by considering a flip in the sign of the magnetic field vector for the Onsager reciprocal coefficients. Casimir [24] gave further examples for departure from Onsager's symmetry condition and highlighted the necessity of making a distinction between even and odd coefficients for the formulation of a general theory. This extension is now known as the Onsager-Casimir reciprocal theorem with $L_{ij}=\pm L_{ji}$ for all i≠j.

Here, we draw attention to the problem that Onsager's original microscopic reversibility theorem strictly leads to macroscopic reversibility [80]. Onsager's reciprocal condition does not offer solutions to the missing link from microscopic reversibility to macroscopic irreversibilty, also known as Loschmidt's paradox. The theorem neglects the fact that e.g.; frictional heating occurs, where mechanical work is converted into heat transfer at macro-scale by shearing of microscopic asperity contacts; or another example is the macroscopic irreversibility of the macroscopic Carnot engine that clearly requests an extension to the formulation [81]. We propose that the additional entropy production of micro-reversible non-local cross-diffusion processes including Casimir's skew symmetric $L_{ij}=-L_{ji}$ provides the key. For simplicity we restrict ourselves to a bipartite system considering just two interacting processes at microscale that co-evolve towards their conjoint maximum entropy production. After discussion of a particular case study we will propose a new thermodynamic reference case for non-local equilibrium where flutter instabilities emerge as a precursor to macroscopic failure.

## 9 Non-local reaction-cross diffusion equations for bipartite media

The difference between our approach and the standard (coarse-grained) approach is accounting for the entropy generation due to the cross-coupled thermodynamic force-flux relations of fine-grained interactions and their phase changes which can introduce a new dynamic time-scale of growing networks as discussed above (Fig. 5). The transformation from coarse-grain to fine-grain configuration therefore does not only contain mapping in space but also mapping in time as will be shown in the following example. In the example we combine the above insights from a coarse grain perspective (classical acceleration waves) and the upscaled hydrodynamic fine-grained approaches (propagation of the active zone) by using a non-local approach to assess the dissipative diffusion and reaction processes. We will show that the approach adds the necessary information of mutual information in terms of cross-diffusional fluxes between the dynamics of the active zone at fine-grained configuration and its interaction with the coarse-grained configuration. We will also show that a new class of dissipative excitation waves with a characteristic spectral content emerges through consideration of this non-locality. The approach allows estimation of conditions for the nucleation, propagation speed and size of active zones from linear stability analysis of the system of equations. This analysis identifies critical conditions where information exchange between the micro- and macro-system occurs via constant velocity dissipative material waves which fall into the category of quasi-soliton waves [82]. They are here called cross-diffusion waves which are identified as an important class of acceleration waves, that occur in the form of a flutter instability prior to the onset of macroscopic failure. The theoretical approach will be illustrated by an applied 1-D example of propagating compaction waves in a porous medium.

### 9.1 Application to the dynamic evolution of a fully saturated porous medium

We have identified the mutual information as the additional entropy source $S_{s,f}^{bi}$ that distinguishes passive matter from active matter. This tight coupling consideration directly leads to the more general cross-diffusion formulation used for describing behaviour of biological, chemical and ecological systems where the concentration/density gradient of one species is influenced by the flux of another species including the option of tight coupling between the species [83].

The literature on cross-diffusion is vast and mathematical modelling has helped to clarify the behavior and stability of the complex system being modelled. The advantage of the approach is a significant simplification of the complex system dynamics where a linear stability analysis can be used to provide an overview over the rich solution space including spectral content of wave instabilities. Here we focus on a special family of solutions for skew symmetric cross-diffusion coefficients which have been well studied and have been identified as a new class of waves called quasi-solitons [73]. Therein, the concern over the “negative entropy” appearing, from a macroscopic continuum perspective, from neglected internal sources of energy of active matter, can also be shown to be related in reduced models to the diffusion part thus obeying the second law of thermodynamics. In these reduced models the bipartite component is simply added as an additional third virtual species with positive entropy production at macro-scale. We discuss in the following why a tightly coupled porous medium witnessing internal mass transfer can be reduced to this skew symmetric form. The discussion also allows us to highlight the role of non-locality in the formulation.

To this end we explicitly consider the mass exchange processes in a fully saturated porous medium using coarse graining by mixture theory [84]. We have introduced the solid and fluid mass fractions *s* and *f* as a tightly coupled state capable of internal mass exchange. For coarse graining their rate of mass transfer ${\overset{˙}{\xi }}_{i}^{REV}$ we consider a large-scale “Representative Elementary Volume” $V_{REV}$ where $V_{s}$, $V_{f}$ are the volumes of the solid and fluid phase, respectively.

(20a) $${\overset{˙}{\xi }}_{i}^{REV}=\frac{1}{V_{REV}}\int_{V_{REV}}{\overset{˙}{\xi }}_{j}^{local}dV_{REV},$$

(20b) $${\overset{˙}{\xi }}_{j}^{REV}=\frac{1}{V_{REV}}\int_{V_{REV}}{\overset{˙}{\xi }}_{i}^{local}dV_{REV},$$

where ${\overset{˙}{\xi }}_{i}^{local}$ and ${\overset{˙}{\xi }}_{j}^{local}$ are the local mass exchange rates from the *s* to *f* phase and vice-versa.

The REV volume fraction of the solid phase is:

(21) $$\phi =\frac{V_{s}}{V_{REV}}=1-\frac{V_{f}}{V_{REV}},$$

Mass conservation at REV scale implies that:

(22a) $$\frac{\partial [\rho_{s}V_{s}]}{\partial t}+\frac{\partial [\rho_{s}V_{s}v_{s}]}{\partial x}={\overset{˙}{\xi }}_{s}V_{REV},$$

(22b) $$\frac{\partial [\rho_{f}V_{f}]}{\partial t}+\frac{\partial [\rho_{f}V_{f}v_{f}]}{\partial x}={\overset{˙}{\xi }}_{f}V_{REV}.$$

$\rho_{s}$ and $\rho_{f}$ identify the density of the solid and fluid phases, while $v_{s}$ and $v_{f}$ their velocities in the direction of *x* and ${\overset{˙}{\xi }}_{s}$ and ${\overset{˙}{\xi }}_{f}$ represent the volume averaged mass generation in the REV.

Substituting Eq. (21) into Eq. (22a), (22b) and eliminating $V_{REV}$ gives

(23a) $$\frac{\partial [\rho_{s}\phi ]}{\partial t}+\frac{\partial [\rho_{s}\phi v_{s}]}{\partial x}={\overset{˙}{\xi }}_{s}^{REV},$$

(23b) $$\frac{\partial [\rho_{f}(1-\phi )]}{\partial t}+\frac{\partial [\rho_{f}(1-\phi )v_{s}]}{\partial x}={\overset{˙}{\xi }}_{f}^{REV}.$$

We apply the same mass balance consideration for the local scale:

(24a) $${\overset{˙}{\xi }}_{f}^{local}=\frac{\partial [\rho_{f}(1-\phi^{local})]}{\partial t}+\frac{\partial [\rho_{f}(1-\phi^{local})v_{f}]}{\partial x},$$

(24b) $${\overset{˙}{\xi }}_{s}^{local}=\frac{\partial [\rho_{s}\phi^{local}]}{\partial t}+\frac{\partial [\rho_{s}\phi^{local}v_{s}]}{\partial x},$$

Coarse graining Eq. (24a), (24b) into Eq. (23a), (23b) through (Eq. (20a), (20b)), we have:

(25a) $$\frac{\partial [\rho_{s}\phi ]}{\partial t}+\underset{Self-diffusion}{\underset{︸}{\frac{\partial [\rho_{s}\phi v_{s}]}{\partial x}}}+\underset{Cross-diffusion}{\underset{︸}{\frac{1}{V_{REV}}\int_{V_{REV}}\frac{\partial [\rho_{f}(1-\phi^{local})v_{f}]}{\partial x}dV_{REV}}}=-\frac{1}{V_{REV}}\int_{V_{REV}}\frac{\partial [\rho_{f}(1-\phi^{local})]}{\partial t},$$

(25b) $$\frac{\partial [\rho_{B}(1-\phi )]}{\partial t}+\underset{Self-diffusion}{\underset{︸}{\frac{\partial [\rho_{B}(1-\phi )v_{B}]}{\partial x}dV_{REV}}}+\underset{Cross-diffusion}{\underset{︸}{\frac{1}{V_{REV}}\int_{V_{REV}}\frac{\partial [\rho_{A}\phi^{local}v_{A}]}{\partial x}dV_{REV}}}=-\frac{1}{V_{REV}}\int_{V_{REV}}\frac{\partial [\rho_{A}\phi^{local}]}{\partial t}dV_{REV},$$

When applying the approach to the case of a dynamic problem in a fully saturated porous medium the entropy production for the solid and fluid phases is characterised by the change in densities ($\rho_{s},\rho_{f}$) as introduced in Eqs. (23a), (23b) and (25a), (25b). The compaction of the solid phase (skeleton) implies mass transfer into the unit volume which must be compensated by outward directed fluid flow. The signs of the $L_{HM}$ and $L_{MH}$ in Eq. (2) must therefore be odd implying that fluid evolution is interpenetrating the matrix of the solid compaction process. Following the dynamic renormalisation approach we propose that a meso-scale equilibrium exists where the maximum entropy of the fluid-solid mass-exchange microprocesses is characterised by the diffusional length scale $l_{d}$. In section 6.1 we have discussed the self-excitation phenomenon of the opposite mutually attracting flows induced by solid and fluid pressure gradients. According to the definition of dense active matter this process must fulfill two criteria: (1) by neglecting the role of temperature we assumed in the above discussed example that the mass flux is significantly larger than thermally induced vibrations; (2) by setting the self-diffusion coefficient to zero we can ensure that the typical persistence time is larger than characteristic relaxation times of the system in the absence of activity. It follows from this interpretation that the phenomenon of cross-diffusion with two skew symmetric $L_{HM}=-L_{MH}$ can form the propagation of an active zone that drives the nucleation of acceleration waves.

(26) $$L_{ij}^{ref}=\left(\begin{array}{cc} 0\; & L_{HM}\\ -L_{HM}\; & 0 \end{array}\right)$$

This matrix is also known as the symplectic matrix of a bipartite system which we propose to be the ‘objective’ reference frame for the bipartite system through its mutual information entropy state. The antisymmetric matrix has been identified as the key element for describing couplings of systems with non-local gain and loss of energy [85]. The lack of reciprocacy was found to lead to instabilities at the exceptional point. We expect, however, that in real-world systems these instabilities are unnoticed as they are halted at larger scale by the necessity to trigger different multiphysics feedback processes with larger diffusion length scales at the macroscopic view of the observer. The potential for global instabilities stemming from exponential growth of long-wavelength perturbations at the EP is, however, even more critical when considering an extension of the matrix to include a higher degree of multimodal and multiphysics processes as discussed next.

The original Onsager Matrix is a Hermitian matrix where the $L_{ij}$ coefficients are relaxing to a non-equilibrium steady state entropy production defined by the real-valued eigendecomposition of the $L_{ij}$ matrix, i.e. the effective self-diffusion coefficients. Here we allow for additional time scale processes defined by the cross-diffusion coefficients and cross-coupled reaction rates where large fluctuations of entropy production are possible.

So far we have discussed just hydromechanical coupling resulting in a 2×2 skew symmetric cross-diffusion symplectic matrix upon adiabatic elimination of self-diffusion coefficients ($L_{ii}=0$). We propose for generalisation a bilinear symplectic subgroup of a general 2n×2n linear group, where *n* corresponds to the number of bipartite microprocesses. For inclusion of more microprocesses listed in Eq. (2) we therefore propose a new skew orthogonal dynamic Onsager Matrix reference frame for cases where coupled multiphysics processes can trigger instabilities from the subcritical regime. We have by now discussed the thermally quenched state were the temperature of the thermal bath is neglected. We may wish to add the thermal fluxes and forces and consider in addition the following symplectic matrix:

(27) $$L_{ij}^{ref}=\left(\begin{array}{cccc} 0 & L_{TH} & 0 & 0\\ -L_{TH} & 0 & L_{TM} & 0\\ 0 & -L_{TM} & 0 & L_{TC}\\ 0 & 0 & -L_{TC} & 0 \end{array}\right)$$

This form defines a bilinear general vector space describing the reference frame of the multiple interacting microprocesses with a symplectic basis equivalent to the orthonormal basis obtained by deriving effective diffusion coefficients $L_{ii}^{ref}$ in equation (2) as eigenvalues of an Euclidean space. This approach is extendable to any amounts of multiphysics couplings. We have illustrated a possible extension of Eq. (27) using a hierarchical tensor network of bipartite THMC couplings as a multiphysics candidate for the physics of the earthquake phenomenon [49].

Williamson [86] discussed the normal form of linear dynamic systems with skew symmetric matrices where the symplectic eigenvalues (diagonal coefficients) are imaginary numbers [87] capturing the bipartite relationship of the network forming process. The complex Euclidean space retains the information of the bipartite definition of the tightly coupled reactions which we propose as a new reference frame when cross-diffusion coefficients become significant. The appearance of complex-valued eigenvalues in Euclidean space follows from the skew symmetry of the network forming processes of the positive and negative entropy production by the micro-engines, each within their own thermal bath. The network forming processes introduce the new system dynamics encapsulating the thermodynamic uncertainty required for the definition of the nonlinear interactions, i.e., beyond the Onsager-Casimir relations.

This is significantly different to the classical Onsager-Casimir assumption of micro-reversibility where the eigenvalues (self-diffusion coefficients) are real-valued. Real-valued eigenvalues describe the unique local equilibrium condition which is only valid if all micro-engines can be upscaled as a single effective medium, without considering the mutual information describing the bipartite disorder information at macroscopic scale. The complex macroscopic bipartite self-organised system captures the paired antisymmetric non-local cross-diffusion coefficients. This may be understood as the upper bound of the entropic uncertainty relationship [7] for the case of non-linear interactions in dense active matter modelled as a bipartite system in complex Euclidean space.

### 9.2 Numerical solution of active zone flutter instabilities

Linear stability analysis of the equations reveals a rich field of instabilities which will be the subject of a forthcoming contribution. Stationary localisation bands are interpreted as a special class of quasi-static instabilities that appear, according to the linear stability analysis, in a critical domain of negative fluid and solid production rates (see Fig. 1 in Ref. [88]). Here we would like to briefly discuss whether flutter instabilities can indeed be resolved as a preparation for the formation of macroscopic instabilities that are interpreted as standing wave instabilities with acceleration wave velocity v=0. For this we consider the nominally stable regime where fluid and production rates are sufficiently small such that quasi-static instabilities are suppressed.

In frictional experiment of this subcritical regime nucleation of flutter instabilities is expected at a critical strain [43]. We note from the findings of active zone dynamics [22] that a nonlinear term (Eq. (11)) in the Langevin equation is required. The reactive source term of the solid pressure reaction-cross-diffusion equation is therefore assumed to be nonlinear by choosing a series expansion of Perzyna overstress [89] creep laws to third order with the rates $a_{11}{\overset{¯}{p}}_{\text{s}}+a_{12}{{\overset{¯}{p}}^{2}}_{\text{s}}+a_{13}{{\overset{¯}{p}}^{3}}_{\text{s}}+a_{14}p_{\text{f}}$. The feedback from the cross-coupled fluid pressure is assumed to be linear and the fluid pressure source term is defined by the sum of the rate of cross-coupled fluid production from the solid and the pressure response from the fluid $a_{21}{\overset{¯}{p}}_{\text{s}}+a_{22}p_{\text{f}}$. We also use a normalisation of the solid and fluid pressure reaction-cross-diffusion (allowing skew symmetric coefficients for tight non-local coupling) by introducing a nondimensional time scale $\overset{˜}{t}={\overset{˙}{\varepsilon }}_{0}t$ defined by a reference strain rate ${\overset{˙}{\varepsilon }}_{0}$. The 1-D spatial coordinate is normalised by the initial length $l_{0}$ of the compacting sample $\overset{˜}{x}=\frac{x}{l_{0}}$. The respective pressures are obtained by introducing reference values for the fluid pressure ${\overset{˜}{p}}_{\text{f}}=p_{\text{f}}/p_{\text{ref}}^{\prime }$ and the overstress of the solid skeleton ${\overset{˜}{p}}_{\text{s}}={\overset{¯}{p}}_{\text{s}}/p_{\text{ref}}^{\prime }$. This leads to following definitions: ${\overset{˜}{L}}_{\text{MM}}=L_{\text{MM}}/{l_{\text{0}}}^{2}{\overset{˙}{\varepsilon }}_{\text{0}}$, ${\overset{˜}{L}}_{\text{HH}}=L_{\text{HH}}/{l_{0}}^{2}{\overset{˙}{\varepsilon }}_{0}$, ${\overset{˜}{a}}_{11}=a_{11}/{\overset{˙}{\varepsilon }}_{0}$, ${\overset{˜}{a}}_{12}=a_{12}p_{\text{ref}}^{\prime }/{\overset{˙}{\varepsilon }}_{0}$, ${\overset{˜}{a}}_{13}=a_{13}{p_{\text{ref}}^{\prime }}^{2}/{\overset{˙}{\varepsilon }}_{0}$, ${\overset{˜}{a}}_{14}=a_{14}/{\overset{˙}{\varepsilon }}_{0}$, ${\overset{˜}{L}}_{\text{HM}}=L_{\text{HM}}/{l_{0}}^{2}{\overset{˙}{\varepsilon }}_{0}$, ${\overset{˜}{L}}_{\text{MH}}=L_{\text{MH}}/{l_{0}}^{2}{\overset{˙}{\varepsilon }}_{0}$, ${\overset{˜}{a}}_{21}=a_{21}/{\overset{˙}{\varepsilon }}_{0}$, ${\overset{˜}{a}}_{22}=a_{22}/{\overset{˙}{\varepsilon }}_{0}$.

The nondimensional reaction-cross-diffusion equation becomes

(28) $$\frac{\partial {\overset{˜}{p}}_{\text{s}}}{\partial \overset{˜}{t}}={\overset{˜}{L}}_{\text{MM}}\frac{\partial^{2}{\overset{˜}{p}}_{\text{s}}}{\partial {\overset{˜}{x}}^{2}}+{\overset{˜}{L}}_{\text{HM}}\frac{\partial^{2}{\overset{˜}{p}}_{\text{f}}}{\partial {\overset{˜}{x}}^{2}}+{\overset{˜}{a}}_{11}{\overset{˜}{p}}_{\text{s}}+{\overset{˜}{a}}_{12}{\overset{˜}{p}}_{\text{s}}^{2}+{\overset{˜}{a}}_{13}{\overset{˜}{p}}_{\text{s}}^{3}+{\overset{˜}{a}}_{14}{\overset{˜}{p}}_{\text{f}},$$

(29) $$\frac{\partial {\overset{˜}{p}}_{\text{f}}}{\partial \overset{˜}{t}}={\overset{˜}{L}}_{\text{HH}}\frac{\partial^{2}{\overset{˜}{p}}_{\text{f}}}{\partial {\overset{˜}{x}}^{2}}+{\overset{˜}{L}}_{\text{MH}}\frac{\partial^{2}{\overset{˜}{p}}_{\text{s}}}{\partial {\overset{˜}{x}}^{2}}+{\overset{˜}{a}}_{21}{\overset{˜}{p}}_{\text{s}}+{\overset{˜}{a}}_{22}{\overset{˜}{p}}_{\text{f}}.$$

**Figure 6:**
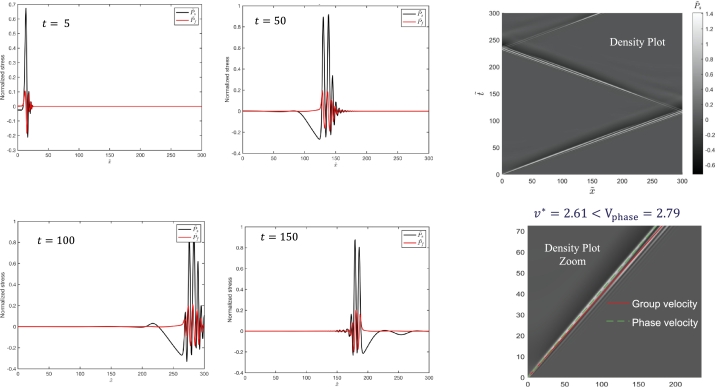
Four time steps of a propagating cross-diffusion wave formed by active zone coupling of solid and fluid transfer processes, here illustrated by the solid pressure (red) and fluid pressure (black). The two pressure waves support each other through mutual excitation and turn a passive poromechanical medium into active matter for following solid pressure production rates: *a*_11_ = −0.05,*a*_12_ = −3,*a*_13_ = 1,*a*_14_ = −1; for the fluid pressure reaction rates we use: *a*_22_ = 0,*a*_21_ = 0.01 regularised by a symplectic cross-diffusion matrix *L*_*MH*_ = −1,*L*_*HM*_ = 1. The right panel shows the density plot of ${\overset{˜}{p}}_{\text{s}}$ which is the longer wavelength of the two pressure waves. Note that after each reflection the amplitude temporarily diminishes and recovers its original magnitude about mid-section. After each reflection the sample has compacted a small fraction due to the passage of the wave at a constant velocity *v*^⁎^ which is bounded by 2*v* and results from a convolution of the solid and fluid pressure diffusion waves illustrated in Fig. 1. The numerical formulation and a link to the open access code can be found in [88]. A video animation of Fig. 6 is available as supplementary material.

We show an example for the nondimensional version of the symplectic Eq. (26) with ${\overset{˜}{L}}_{MH}=-1,{\overset{˜}{L}}_{HM}=1$. The resulting cross-diffusion wave is shown in Fig. 6 which illustrates the moving objective reference frame of the cross-diffusion wave that propagates with a group velocity $v^{⁎}$. The density plot identifies $v^{⁎}$ as the constant wave group velocity (=2.61) of the cross-diffusion wave which is internally composed of slightly faster phase velocities (≥2.79 corresponding to the solid pressure wave). The group velocity corresponds to the velocity of the background entropy production, which we interpret as a fundamental material property of the fluid saturated porous medium. The group velocity $v^{⁎}$ of the cross-diffusion waves therefore is not dependent on boundary conditions.

The group velocity is expected to be slower than the upper bound velocity of the propagation of the active zone $v^{⁎}<2v$ for cases of non-zero self-diffusion coefficients. The reason for this is that the propagation entropy of the active zone is greater than the mutual information entropy, because in a realistic case we have to consider in addition to the mutual information between the two phases coupling to the coarse grain environment. The presence of additional correlations that are caused by the interaction with the self-diffusing environment is therefore expected to slow down the speed of the propagating wave.

Note that after each reflection the microstructure is expected to have changed after passage of the compaction wave leaving in its wake a certain reduction in porosity (not considered here). Quantifying the effect of continuous compaction on the change of the cross-diffusion coefficients will be an interesting follow-up study. We expect an increased wave speed upon reflection as the topology of the microstructure has changed and the porosity and permeability has reduced leading to a tighter environment and a reduction of the diffusive length scale $l_{d}$.

If we assume that the rheology of the solid matrix remains unchanged by passage of the waves a reduced permeability brings the system closer to the critical condition [25], [88] for the onset of the stationary compaction band solution. We therefore propose that the incremental compaction of the sample changes the cross-diffusion matrix towards the critical parameters that are needed to form the standing wave solution of the stationary localisation band described above.

## 10 Discussion

In this contribution we have approached the phenomenon of the formation of localisation bands for the simple case of a 1-D instability. The approach is based on an extension of Onsager's seminal relationship for cases where the symmetry condition is broken and the cross-diffusion terms play a significant role in the evolution of the localisation band. We have identified the essential role for nucleation of dissipative cross-diffusion waves in the case of skew symmetric coefficients missing in the original formulation of Onsager's reciprocal theorem. The completeness of Onsager relations is hence questioned in the context of multiphysics induced dynamic instabilities across process-defined scales in a bipartite system, in particular. On the mathematical side, the importance of complex eigenvalues of the Onsager matrix for nucleation of cross-diffusion waves was first identified in Vanag [83].

We have started the analysis with the question on how our new findings relate to the fundamental relationship of the acoustic tensor criterion for localisation [38] that has been tested over and over in the laboratory and forms the backbone of the phenomenon of localisation bands in porous materials for nearly fifty years. Our approach converges to the quasistationary case for $v^{⁎}=0$ and simply extends the quasistatic approach into the dynamic solution space. In fact, Hill [36] has set the ground works for both static and dynamic approaches in his classical work on acceleration waves in solids. The relation of the acoustic tensor criterion for the limiting case of zero velocity acceleration waves has been discussed in Rice [27] and the relationship to the theory presented in Ref. [62]. This common origin provides an ideal ground for further developments and will be the subject of future work on developing methods for forecasting earth instabilities and designing new practices for sustainable energy and mineral resources.

We propose that our dynamic perspective of localisation phenomena allows new insights into precursor phenomena for macroscopic failure. In this respect the emergence of cross-diffusion waves, which we have related to the phenomenon of flutter instabilities in the macroscopic theories, may provide the key to recording precursors for failure [60], [41]. We propose that these waves should be measurable in the laboratory and may have already been recorded in experiments on crushed snow [90]. As the characteristic background velocity $v^{⁎}$ is interpreted to be a material constant such laboratory experiments performed on multiple materials may be used for creating a material data base for the detection of cross-diffusion waves in natural and engineering structures. Another approach is to use geophysical time series data of recorded time-periodic instabilities to derive coefficients of the reaction-cross diffusion equations which has been performed in a first pilot study [88] and is part of ongoing work.

As a final comment the work is not limited to conventional porous materials and opens up new links to recent discoveries following the path on nonlinear dynamics of bipartite systems and emergence of active matter with significant future potential for other related applications where small scale internal mass-exchange processes are an important driver of macroscopic phenomena.

## 11 Conclusions and future outlook

The present work sets out to understand multiscale and multiphysics instabilities in nature from a fundamental physics-based perspective. The commonality of reaction-cross-diffusion feedbacks across vast scales from the nanometer level to the large geodynamic scale of plate tectonics requires a new basis explaining the self-similarity of instabilities across vast length and time scales.

There are three challenges. First, the underlying coupled thermal, hydro-mechanical and chemical, (THMC) multi-physics processes are complex and often poorly understood. Second, the solution of the coupled systems of partial differential equations is mathematically and computationally challenging, and third, direct microstructural observations of dynamic processes in the deforming porous medium are occluded to the observer.

To solve these challenges, we have proposed the present new non-local multi-physics approach. The approach extends the classical Onsager reciprocal theorem which formulates a real value eigenvalue problem for the dissipation of multiscale and multiphysics problems (when cast into a THMC matrix) by a complex value eigenvalue problem for non-local cross-diffusion interactions when the role of microstructure becomes an important ingredient. This enables investigation of precursor mechanisms to large scale instabilities that have so far been overlooked in geo-processes, that is, the potential release of energy stored within the microstructure of solids by activating micro-macro-scale feedbacks.

Extensive recent studies, mostly experimental, on active matters in biological, biopolymers, colloidal systems, and artificial particles have been paving the way. We have highlighted that dense active matter, driven far from equilibrium on its microscale, displays unexpected behaviour by non-local dissipation of internal energy releasing its dynamic incompatibility with the macroscopic gradients as acceleration waves under external forcing. When thermal forces, that can obliterate directional mass movement by these processes, are insignificant it can lead to the propagation and reflection of quasi-soliton excitation waves. Under the right conditions can an external load sustain a “perpetuum-mobile-like” behaviour. For a constant load, while internal (microstructural) energy is available, the motion continues; otherwise, it stops. These phenomena are commonly classified as “active matter” and recently proposed to bring together the physics of glass, jamming, plasticity and turbulence, in a new state of driven classical matter [55].

Dense active matter involves chemical or fluid and mechanical mass fluxes to be unbalanced locally such that no effective thermodynamic force principle applies. It allows consideration of the possible solid pressure fluxes through matrix deformation with fluid diffusion through non-local diffusive regions. This interdependence can cause the local fluid pressure to go through rapid changes accompanied by enforced mass exchange within the matrix environment in the highly pressurized mixture [67]. Such systems are also understood as active matter systems in which classical physics fails to describe the additional capacity to tunnel through an energy barrier by activating non-local interactions. It is proposed here that the common principle applies to connect processes at and across their characteristic scales.

The choice of a 1-D configuration enables the use of scalar variable field variables of solid and fluid pressures. This enables a minimalist analytical treatment of the expected instabilities through linear and nonlinear stability analysis. The simple formulation has also allowed merging insights gained from diffusion-relaxation of dynamical optimal transport problems [69] and active zone dynamics [22] including active matter physics [55] to the well established theories of solid mechanics on acceleration waves and flutter instabilities [36], [27], [38]. Future work will need to compare model predictions to carefully designed laboratory experiments and expand the work to bridge the scale of the laboratory to the scale of field applications through coupling our theoretical and numerical modelling work, transforming our 1D solvers into 3D solvers, and extend the theory from dissipative pressure to dissipative shear waves. We have presented a multiphysics generalisation of the phenomenon to the exciting findings of fluctuations with oscillatory exponential growth which nucleate at the exceptional point for inception of complex conjugate eigenmodes (flutter instabilities) in frictional materials [43]. In this sense experiments with granular matter, as an archetype dense active matter, promises testing and advancing the theory presented here. Ongoing and future work will tackle the extension of the approach to intertwine candidate symplectic matrices of the style of Eq. (27) with additional THMC-combinations, building candidate tensor networks that enable global percolation of instabilities from atomic to plate tectonic scale. Such an approach could assist in revealing the elusive physics driving the hierarchy of multiscale material instabilities that precede and lead to the earthquake phenomenon.

## CRediT authorship contribution statement

**Klaus Regenauer-Lieb:** Writing – review & editing, Writing – original draft, Validation, Supervision, Resources, Project administration, Methodology, Investigation, Funding acquisition, Formal analysis, Conceptualization. **Manman Hu:** Writing – review & editing, Validation, Supervision, Methodology, Investigation, Funding acquisition, Formal analysis, Conceptualization.

## Declaration of Competing Interest

The authors declare that they have no known competing financial interests or personal relationships that could have appeared to influence the work reported in this paper.

## Data Availability

No data was used for the research described in the article.

## Appendix A Supplementary material

Video Video animation of Fig. 6. Video
